# Modulation of Gut Microbiota by Glucosamine and Chondroitin in a Randomized, Double-Blind Pilot Trial in Humans

**DOI:** 10.3390/microorganisms7120610

**Published:** 2019-11-23

**Authors:** Sandi L. Navarro, Lisa Levy, Keith R. Curtis, Johanna W. Lampe, Meredith A.J. Hullar

**Affiliations:** 1 Division of Public Health Sciences, Fred Hutchinson Cancer Research Center, Seattle, WA 98109, USA; llevy@fredhutch.org (L.L.); krcurtis@fredhutch.org (K.R.C.); jlampe@fredhutch.org (J.W.L.);; 2 Department of Epidemiology, University of Washington, Seattle, WA 98195, USA

**Keywords:** gut microbiome, glucosamine, chondroitin, crossover trial

## Abstract

Glucosamine and chondroitin (G&C), typically taken for joint pain, are among the most frequently used specialty supplements by US adults. More recently, G&C have been associated with lower incidence of colorectal cancer in human observational studies and reduced severity of experimentally-induced ulcerative colitis in rodents. However, little is known about their effects on colon-related physiology. G&C are poorly absorbed and therefore metabolized by gut microbiota. G&C have been associated with changes in microbial structure, which may alter host response. We conducted a randomized, double-blind, placebo-controlled crossover trial in ten healthy adults to evaluate the effects of a common dose of G&C compared to placebo for 14 days on gut microbial community structure, measured by 16S rRNA gene sequencing. Linear mixed models were used to evaluate the effect of G&C compared to placebo on fecal microbial alpha and beta diversity, seven phyla, and 137 genera. Nine genera were significantly different between interventions (False Discovery Rate < 0.05). Abundances of four *Lachnospiraceae* genera, two *Prevotellaceae* genera, and *Desulfovibrio* were increased after G&C compared to placebo, while *Bifidobacterium* and a member of the *Christensenellaceae* family were decreased. Our results suggest that G&C affect the composition of the gut microbiome which may have implications for therapeutic efficacy.

## 1. Introduction

Glucosamine and chondroitin (G&C) are among the most frequently used specialty supplements by US adults [1]. Typically combined and taken together as a single pill, G&C are commonly used for joint-related osteoarthritis (OA) pain. More recently, several large, prospective cohort studies have shown that use of G and/or C are associated with a reduction in colorectal cancer (CRC) risk [2,3,4,5]. Administration of G has also been shown to improve inflammatory bowel disease in both animal models [6] and humans [7], and the combination of G&C reduced systemic inflammation in a small trial of healthy adults [8]. However, beyond dampening inflammation, little is known about the potential effects of G&C on colon-related physiology in humans.

G is an amino sugar, and C is a glycosaminoglycan (GAG) composed of repeating disaccharide motifs containing sulfate groups and a terminal *N*-acetylgalactosamine group. G&C are poorly absorbed in the upper gut [9], and therefore provide rich substrate for microbial metabolism in the colon. A few recent small-scale studies have evaluated G and/or C and the gut microbiome in humans and have reported changes in microbial abundances [9]. Three studies assessed fecal microbial metabolism of C in vitro [10,11,12]. Only one study was conducted in vivo, and found decreased trends in abundances of *Staphylococcus, Enterococcus* and *Clostridium* in OA patients after oral administration of G [13]. These observations suggest that the microbial metabolism of G&C may alter the gut microbial community. Interindividual variation in gut microbiome composition and metabolic efficiency may also be a factor in explaining the variation of G&C therapeutic efficacy. Therefore, the microbiota may be an important consideration in the context of OA, and have implications for inflammation-related effects [14].

To date, no placebo-controlled trials have been conducted evaluating G and/or C on modulation of gut microbial community structure. Our aim in the present pilot feasibility study was to evaluate the effects of supplementation with a common oral dose of combined G&C compared to placebo for 14 days on gut microbial community modulation in ten healthy adults.

## 2. Materials and Methods

### 2.1. Study Design

**Figure 1:**
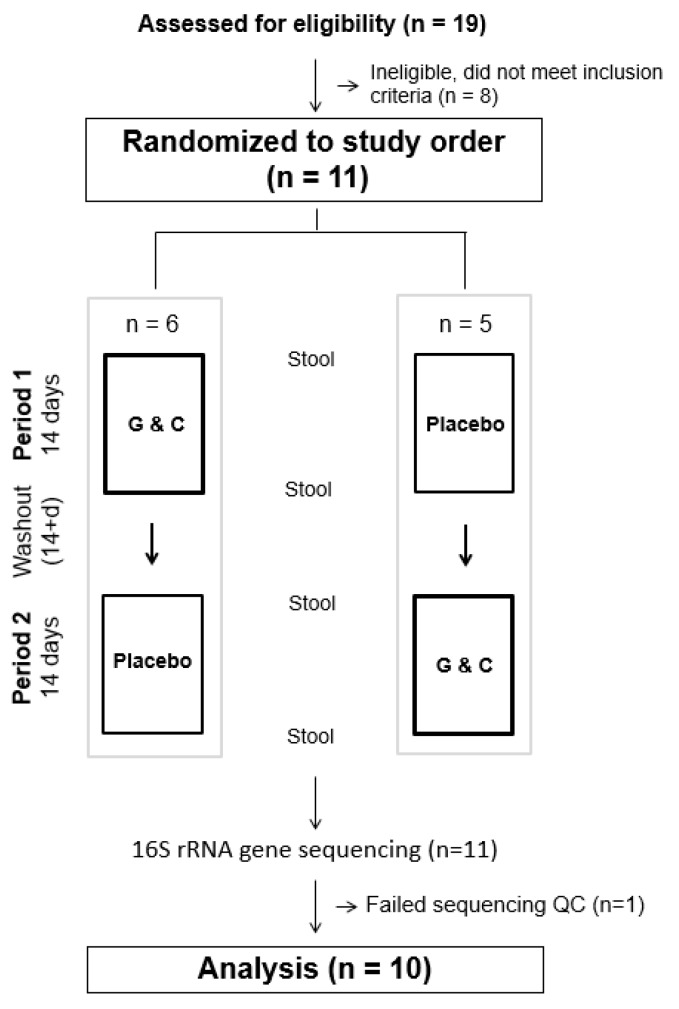
CONSORT figure of the randomized, double-blind, placebo-controlled crossover trial comparing supplemental glucosamine and chondroitin (G&C) to placebo.

The study was a randomized, double-blind, placebo-controlled crossover trial comparing supplemental G&C to placebo. As this was a pilot feasibility trial with the aim of enrolling a sample of 10 individuals, participants were alternately randomized to begin with either the active or placebo intervention (Figure 1). Each intervention lasted 14 days with a minimum of a 14-day washout period between the two interventions. All study activities were carried out at the Fred Hutchinson Cancer Research Center (Fred Hutch), Seattle, WA, in accordance with the Declaration of Helsinki of 1975. Recruitment, enrollment, trial, and sample collection took place from September 2017 to October 2018. The study protocol was approved by the Fred Hutch Institutional Review Board, registered in ClinicalTrials.Gov (NCT03827161 and NCT03963323), and all participants provided written informed consent.

### 2.2. Participants

Participants were healthy, non-smoking, aged 20–50 years, recruited from the greater Seattle area. Exclusion criteria included: chronic illness, history of gastrointestinal, hepatic, or renal disorders, or inflammatory conditions, pregnancy or lactation, currently on a weight-loss diet, alcohol intake > 2 drinks/d, current use of prescription or over-the-counter medications [other than oral contraceptives, multivitamins or infrequent use of aspirin and non-steroidal anti-inflammatory drugs (NSAIDS) < 2 days/week], abnormal renal, liver, or metabolic laboratory values, known allergy to shellfish, or any antibiotic use within the past six months. Participants were asked to abstain from taking multivitamins, aspirin, or NSAIDS, and maintain their usual physical activity levels during the course of the trial.

Prospective participants attended a screening clinic visit at the Fred Hutch Prevention Center. Height and weight were measured, and blood was drawn in the morning after a minimum of a 12-h. overnight fast, and was used for analysis of renal, liver, and metabolic function, as described previously [8]. Individuals with normal laboratory values were invited to participate in the study. A total of 11 individuals were randomized and completed the two intervention arms.

### 2.3. Glucosamine Hydrochloride and Chondroitin Sulfate Supplements

The active treatment (Cosamin^®^ DS) contained 1500 mg/d glucosamine hydrochloride (FCHG49^®^, 100% purity) + 1200 mg/d sodium chondroitin sulfate from bovine cartilage (TRH122^®^, 98% purity), a glycosaminoglycan consisting of alternating d-glucuronic acid and *N*-acetyl-d-galactosamine with sulfation occurring at either the 4 or 6 position, taken as 3 capsules daily with each capsule containing 500 mg glucosamine hydrochloride and 400 mg chondroitin sulfate. Crystalline cellulose was used as an inactive filler, and both active treatment and placebo were encapsulated in clear gelatin capsules. The placebo was similar in appearance and contained only the inactive filler. Both G&C and the placebo were generated from a single lot of source materials and donated by Nutramax Laboratories Consumer Care, Inc. (Edgewood, MD, USA), and were the same products used in our previous study [8]. Supplement bottles were provided by the manufacturer with a single printed label containing the letters “A” or “B”. To maintain double-blinding for both participants and investigators, the randomization algorithm was sent in a sealed envelope; unblinding of the interventions was done after the completion of data analysis. Participants were contacted by study staff at the mid-point and end of each intervention period to assess any possible adverse experiences and compliance. No adverse events were reported. Extra pills were included in each supplement bottle in the event that a Day 14 study visit was delayed, and so that adherence percentages (pills supplied-pills returned/days elapsed) could be calculated as a compliance measure. Based on these metrics, mean (SD) compliance was 97% (±4%) for G&C and 94% (±5%) for placebo. Composition of analysis testing indicated that content of G&C was within required specifications, i.e., 97% of labeled amount for G and 103% for C. No G&C was detected in the placebo capsules and no microbial products were detected in either capsule type.

### 2.4. Stool Collection

Participants collected stool samples at the beginning and end of each intervention period into RNAlater for bacterial measures using a fecal collection tube with a scoop in the lid (Sarstedt, Numbrecht, Germany) containing 5 mL preservation solution and 8–10 glass beads (3 mm; Fisher, Waltham, MA, USA) [15]. They were instructed to collect 2 pea-sized aliquots of stool and immediately, at the time of defecation, place the stool into the collection tube and mix well by shaking. The samples were delivered to the laboratory within 24 h and stored at −80 °C.

### 2.5. Fecal Microbiome Measures

Stool samples collected in RNAlater were thawed and homogenized, and DNA was extracted and amplified and sequenced for the V4 region of the 16S rRNA gene, as described previously [16]. Paired-end sequencing was performed on the MiSeq using MiSeq Reagent Kit v3 following the manufacturer’s guidelines to obtain 2 × 300 bp paired-end reads (Illumina, San Diego, CA, USA). FastQ files were exported (Molecular Research, Shallowater, TX, USA) and securely transferred to Fred Hutch (BaseSpace, Illumina, San Diego, CA, USA) for bioinformatic analysis.

### 2.6. Microbiome Bioinformatic Analysis

To classify bacterial taxonomy, sequences were processed using QIIME v.1.8 [17] using the SILVA database (release 132, clustered at the 97% similarity level) [18] as previously described. Sequence counts for each sample ranging from phylum to genus level were generated on unrarefied data. Alpha diversity measures [Shannon index [19]], beta diversity matrices [unweighted and weighted UniFrac [20,21]] using counts of sequences were exported for statistical analysis.

### 2.7. Statistical Analysis

Eleven participants were randomized and completed all study activities. The stool samples from one participant did not pass our stringent sequencing parameters [16] leaving a total of 10 participants, 3 men and 7 women, for statistical analysis. Files of sequence counts were used in statistical analysis. Genera with >0.5% prevalence were included in the analyses. To account for the compositional nature of the microbial abundances, we calculated the centered log-ratio (CLR) transformation [22]. For this purpose, a pseudo-count of 1 was added to the raw sequence count for each taxon prior to transformation. We used permutational multivariate analysis of variance (PERMANOVA) to assess the effect of G&C compared to placebo on overall microbial community structure [23]. We tested the effect of G&C intervention compared to placebo on alpha (Shannon–Weaver) diversity using linear mixed models that accounted for the repeated sampling crossover design. Baseline measures of microbial outcomes and treatment sequence were included as covariates in all models. We evaluated the effects of age, sex, body mass index (BMI), treatment sequence, and baseline microbial measures as covariates. Only BMI and baseline measures had ≥ 10% contribution to point estimates and were included in the models. To ensure that changes in microbial communities were not occurring with both interventions, changes in gut microbiome genera from baseline to end of the placebo only were evaluated in a post-hoc analysis. The Benjamini–Hochberg algorithm [24] was applied to all analyses to control for multiple testing. We also evaluated baseline microbiome measures by treatment period using a Student’s t-test and found that they did not differ, confirming that carryover effects were not contributing to outcome measures (*p* > 0.2 for all tests). Analyses were performed using Stata statistical software (v16, StataCorp, College Station, TX, USA).

## 3. Results

Characteristics of the ten study participants stratified by sex are given in Table 1. Women tended to be older and have a lower BMI than men.

**Figure 2:**
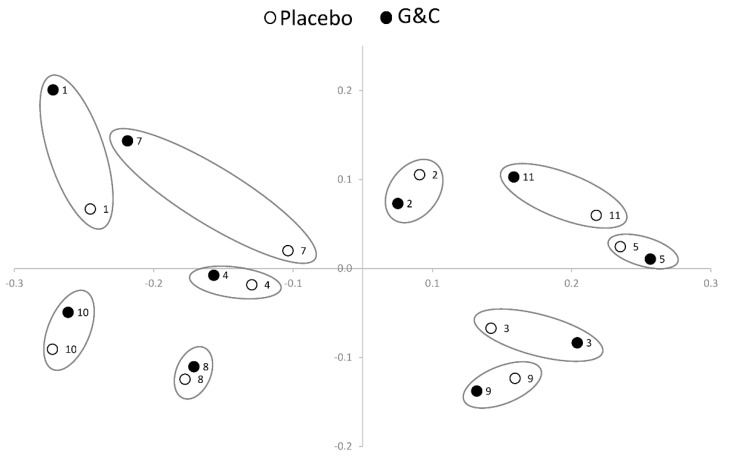
Permutational multivariate analysis of variance (PERMANOVA) plot using unweighted UniFrac sequence counts illustrating the variation in interindividual response of the gut microbiome to supplemental glucosamine and chondroitin (G&C) compared to placebo (*n* = 10). Within-person end of placebo (white circle) and end of G&C intervention (black circle) gut microbiome samples are circled. By way of example, the difference in the microbiome across treatments is larger in participant #7 than in participant #8.

Mean alpha diversity measurements did not differ between the treatments (6.45 ± 0.31 and 6.39 ± 0.32 for G&C and placebo, respectively); however, there was a significant difference in the overall microbiome community structure [*p* < 0.05, permutational multivariate analysis of variance (PERMANOVA)] after G&C compared to placebo. Figure 2 shows a range of interindividual response in unweighted UniFrac sequence counts of the microbiome to the G&C intervention. For example, the change in the microbiome across treatments is larger in participant #7 than participant #8. There were no significant differences between interventions in the seven phyla measured: *Actinobacteria*, *Bacteroides*, *Cyanobacteria*, *Firmicutes*, *Proteobacteria*, *Tenericutes*, and *Verrucomicrobia* [False Discovery Rate (FDR) > 0.05]. Of the 137 genera measured, 33 were nominally significant at *p* < 0.05 between interventions (Table S1), with nine passing the FDR threshold < 0.05 (Table 2). All but two genera were more abundant after G&C compared to placebo. When evaluating the change in genera from pre- to post-intervention for placebo only, there were no genera significant, even at a nominal *p* < 0.05.

## 4. Discussion

In this randomized, double-blind, crossover trial, we found significant differences in nine bacterial genera in response to G&C supplementation in ten healthy adults. Seven taxa, including *Anaerostipes, Lachnospira* and two other uncultured *Lachnospiraceae* genera, two *Prevotellaceae* genera, *Alloprevatella* and *Paraprevotella*, and *Desulfovibrio* were higher after two weeks of oral administration of G&C compared to placebo, while *Bifidobacterium* and a genera in the *Christensenellaceae* family were lower. Additionally, the degree of the overall change in the community structure varied significantly among participants, although alpha diversity did not differ.

Besides the purported impact on OA, there is growing evidence to suggest beneficial effects of G&C use on other inflammation-associated health conditions, including reduced risk of CRC, and possibly cardiovascular disease [3,4,25,26,27]. Some of these associations may be linked to microbial metabolism of G&C that leads to reduced systemic inflammation or production of metabolites that alter other signaling pathways in the host. For example, Sicard et al. reported that G reduced biofilm formation of invasive *E. coli* strains by interfering with its adhesion to epithelial cells and subsequent colonization [28]. As intestinal mucin-derived sugars act as a barrier between the epithelium and microbes, G may also play a role in other host-immune interactions. Indeed, administration of G has been shown to improve inflammatory bowel disease in both animal models [6] and humans [7]. However; while clinically beneficial effects have been observed with both G and C, and there is evidence of broad usage of G by bacteria [29], the majority of research in the context of G&C and the gut microbiome pertains to C.

C is a sulfated GAG made up of a polymer of alternating sugars (*N*-acetylgalactosamine and glucuronic acid). Desulfation of C is associated with increased exposure to hydrogen sulfide (H_2_S) [30]. In our study, *Desulfovibrio*, a sulfate reducer, was enriched with G&C supplementation. H_2_S, in a concentration dependent manner, can be either genotoxic or act as a regulatory compound [31]. Deplancke et al. [32] showed in mice that sulfate added to drinking water increased production of H_2_S and resulted in genotoxic effects on the colonic epithelium. In contrast, Rey et al. [33] demonstrated that increases in sulfate-reducing bacteria and colonic H_2_S following C supplementation did not compromise the gut epithelium in gnobiotic mice. Authors also found enhanced production of G from C when sulfate reducers were present [33]. Another recent study in mice suggested that at low concentrations, H_2_S can act as a signal transmitting molecule in metabolic hormone regulation [31]. In particular, Pichette et al. showed that H_2_S, acting through mitogen-activated protein kinase, directly stimulated the incretin, glucagon-like peptide-1 (GLP-1) secretion, and enhanced insulin sensitivity, improved oral glucose tolerance, and reduced food consumption in a mouse model [31]. Whether GLP-1 activation occurs in humans in response to C is unknown.

While most studies of C degradation have shown an enrichment of Bacteroides spp [11,12,34,35], we found an enrichment of genera in *Prevotellaceae*, namely *Paraprevotella* and *Alloprevotella*. C-degrading capability is common within the Bacteroidetes and metabolic specialization for different isomers is linked to genomic polysaccharide utilization loci (PUL). PUL include genes that code for carbohydrate active enzymes, response regulators, and transporters that are involved in the efficient degradation of a specific polysaccharide substrate. Substrate specialization is mirrored in the PUL repertoires resulting in widely heterogeneous and distinct adaptations with regard to the number, source and nature of substrates preferred for growth. These enzymes are involved in the partial extracellular degradation of polysaccharides prior to their transport into the periplasm for final degradation [36]. A recent study showed that variation in PUL enhanced niche specialization in the *Prevotella*. In support of our findings, in *Alloprevotella rava*, the majority of the putative enzyme gene sequences were for PUL involved in GAG degradation [37].

Butyrate, a short chain fatty acid (SCFA), has a positive impact on gastrointestinal tract homeostasis, as it promotes the growth of intestinal epithelial cells, increases the expression of tight junction proteins, and acts as an anti-inflammatory agent [38,39,40,41,42]. In our study, we found a significant increase in genera associated with SCFA-production including *Anaerostipes*, *Lachnospira*, and other uncultured *Lachnospiraceae*. Others have shown enrichment in butyrogenic bacteria associated with increased fecal butyrate after C enrichment [10,30]. Our data also suggest that several different pathways of butyrate production may be enriched in C disaccharide fermentation [43]. *Anaerostipes* produces butyrate directly via acetyl CoA. In contrast, *Lachnospira* produces acetate and indirectly contributes to butyrate production via the interconversion and condensation reactions of two moles of acetate to butyrate. These multiple pathways for bacterial production of butyrate ensure optimal butyrate availability to the host and may positively influence gut homeostasis. For example, a C intervention study showed that circulating markers of inflammation were inversely associated with fecal SCFA in conjunction with decreased circulating levels of lipopolysaccharide (LPS) from gut bacteria, and reduced activation of toll-like receptor (TLR) four signaling [44]. This suggests that one microbial mediated effect of butyrate produced from C fermentation is reduced translocation of LPS and subsequent expression of NF-κB-based inflammation mediators. Several in vitro studies have demonstrated reduced activation of NF-κB with G and/or C [45,46,47]. Further, we reported previously that C-reactive protein, a target of NF-κB regulation, was down regulated in response to G&C in an intervention study in healthy adults [8].

Finally, we found that *Christensenella* and *Bifidobacteria* were lower after the G&C treatment, although other studies have shown that these taxa are involved in GAG degradation [10,44] and that they play a beneficial role in human health [48]. *Christensellaceae*, a recently described family [49], often co-occurs with *Methanobrevibacter* [50] and together they produce SCFAs from GAGs, starch, chitin [51], and hemicellulose [52]. *Bifidobacteria* specialize in the degradation of dietary and host glycan and specialization allows co-existence in the gut microbiome. Our results suggest that other GAGs were also available, i.e., from participant diet, and were the preferred substrates for *Christensellaceae* and *Bifidobacteria* during the G&C intervention.

This is the first randomized, placebo-controlled trial to evaluate the effects of combined G&C on the gut microbiome in healthy adults. A major strength of this study is the crossover design, which allows each person to act as his or her own control, minimizing potential confounding factors. Limitations of the study include the small sample size, and insufficient power for subgroup analyses by sex or adiposity. However, despite the small sample size, the intervention effects of G&C were highly significant, even after controlling for multiple testing. We used 16S rRNA gene sequencing to identify the composition of the microbiome. This approach limits the capacity to identify species and to identify the microbial metabolic genes likely involved in G&C metabolism. For example, two human symbionts, *B. thetaiotamiocron* and *B. ovatus* share 96.5% similarity in their 16S rRNA gene sequence [36]; however, their PUL vary widely, which has enabled them to specialize in the metabolism of different glycan types allowing for niche separation within the gut ecosystem. Future studies warrant the use of metagenomic and in-vitro approaches in combination with our robust intervention design to understand the role of interindividual gut microbial variation in G&C degradation.

## 5. Conclusions

In conclusion, we report that G&C significantly changed the gut microbial community structure and abundance of specific bacterial genera as compared to placebo. These results suggest that microbial metabolism of G&C results in shifts in the gut microbial structure and the production of secondary metabolites that reduce inflammation. This supports previous reports of beneficial effects of G&C on inflammation. Future studies in larger samples and other populations are needed to explore the microbially-mediated mechanisms of action of G&C and understand the influence of interindividual variation in the composition and metabolism of the microbiome on G&C exposure and efficacy.

## Figures and Tables

**Table 1:** Characteristics of study participants stratified by sex, presented as mean (SD) unless otherwise noted.

Parameter	Men (*n* = 3)	Women (*n* = 7)
Age (yrs)	29 (4)	34 (11)
Body mass index (kg/m^2^)	24.1 (4.1)	22.8 (2.5)
Minority, n (%)	2 (67)	2 (29)
Dietary Intakes		
Energy (kcal/d)	2451 (727)	1400 (436)
Carbohydrate (g/d)	266 (41)	148 (37)
Fat (g/d)	101 (38)	60 (24)
Protein (g/d)	115 (52)	64 (24)
Carbohydrate (% of energy)	44.5 (5.8)	43.5 (6.6)
Fat (% of energy)	36.5 (3.1)	38.2 (4.5)
Protein (% of energy)	18.2 (2.7)	17.9 (2.8)
Dietary fiber (g/1000 kcal/d)	11 (2)	13 (3)
Fruit intake (servings/d)	1.3 (0.6)	1.2 (0.7)
Vegetable intake (servings/d)	2.6 (1.7)	2.8 (1.7)
Whole grains (ounce equivalents/d)	2.7 (0.5)	1.3 (1.9)
Alcohol (g/1000 kcal/d)	4 (4)	3 (3)

**Table 2:** Genera significantly different between glucosamine (G) and chondroitin (C) and placebo at day 14.

		Abundance				
	Prevalence	G&C	Placebo	Β ^a^	SE ^a^	*p* Value ^a,b^
**HIGHER after G&C supplementation vs. placebo**						
Firmicutes; Clostridia; Clostridiales;						
*Lachnospiraceae*; *Anaerostipes*	100%	1.6 (0.8)	1.1 (0.6)	0.68	0.18	0.0001
*Lachnospiraceae*; *Lachnospira*	100%	2.8 (2.3)	1.6 (1.4)	0.72	0.23	0.002
*Lachnospiraceae*; *Lachnospiraceae_UCG-001*	100%	0.7 (0.7)	0.4 (0.3)	0.55	0.19	0.003
*Lachnospiraceae*; *uncultured*	100%	1.7 (1.2)	1.1 (0.7)	0.59	0.15	6.55 × 10^−5^
Proteobacteria; Deltaproteobacteria; Desulfovibrionales						
*Desulfovibrionaceae Desulfovibrio*	10%	0.3 (1.1)	0.2 (0.6)	0.28	0.10	0.004
Bacteroidetes; Bacteroidia; Bacteroidales;						
*Prevotellaceae*; *Alloprevotella*	10%	0.1 (0.2)	0.1 (0.3)	0.39	0.13	0.001
*Prevotellaceae*; *Paraprevotella*	30%	0.1 (0.2)	0.2 (0.4)	0.25	0.08	0.0001
**LOWER after G&C supplementation vs. placebo**						
Actinobacteria; Actinobacteria; Bifidobacteriales;						
*Bifidobacteriaceae*; *Bifidobacterium*	100%	2.2 (2.5)	2.8 (3.5)	−0.69	0.21	0.0006
Firmicutes; Clostridia; Clostridiales;						
*Christensenellaceae*; *Christensenellaceae_ R-7_group*	100%	1.2 (1.4)	2.1 (2.4)	−1.22	0.43	0.005

^a^ Beta coefficient (standard error) and *p* values from linear mixed models evaluating the effect of G&C versus placebo on genera, adjusted for treatment sequence and participant body mass index. ^b^ All results presented are significant with False Discovery Rate < 0.05 for 137 tests.

## Supplementary Materials

Supplementary materials can be found at https://www.mdpi.com/2076-2607/7/12/610/s1.
