# Supplementary material for: Modulation of Gut Microbiota by Glucosamine and Chondroitin in a Randomized, Double-Blind Pilot Trial in Humans
Source: Microorganisms. 2019 Nov 23;7(12):610. doi: 10.3390/microorganisms7120610 (PMC6956221; doi:10.3390/microorganisms7120610)
Supplement: Supplementary file 1 [file microorganisms-07-00610-s001.pdf]

microorganisms

MDPI

Supplemental Table 1 - Genera significantly different between glucosamine (G) and chondroitin (C) and placebo at day 14 at P<0.05.

		Abundance		Beta		
	Prevalence	G&C	Placebo	Coef*	SE	P value*
Bacteria;__Firmicutes;__Clostridia;__Clostridiales;__Lachnospiraceae;__uncultured	100%	1.7 (1.2)	1.1 (0.7)	0.59	0.15	6.55E-05
Bacteria;__Firmicutes;__Clostridia;__Clostridiales;__Lachnospiraceae;__Anaerostipes	100%	1.6 (0.8)	1.1 (0.6)	0.68	0.18	0.0001
;__Actinobacteria;__Actinobacteria;__Bifidobacteriales;__Bifidobacteriaceae;__Bifidobacterium	100%	2.2 (2.5)	2.8 (3.5)	-0.72	0.21	0.0006
Bacteria;__Bacteroidetes;__Bacteroidia;__Bacteroidales;__Prevotellaceae;__Paraprevotella	30%	0.1 (0.2)	0.2 (0.4)	0.25	0.08	0.0009
Bacteria;__Bacteroidetes;__Bacteroidia;__Bacteroidales;__Prevotellaceae;__Alloprevotella	10%	0.1 (0.2)	0.1 (0.3)	0.39	0.12	0.0011
Bacteria;__Firmicutes;__Clostridia;__Clostridiales;__Lachnospiraceae;__Lachnospira	100%	2.8 (2.3)	1.6 (1.4)	0.76	0.24	0.0019
Bacteria;__Firmicutes;__Clostridia;__Clostridiales;__Lachnospiraceae;__Lachnospiraceae_UCG-001	100%	0.7 (0.7)	0.4 (0.3)	0.55	0.19	0.0032
Bacteria;__Proteobacteria;__Deltaproteobacteria;__Desulfovibrionales;__Desulfovibrionaceae;__Desulfovibrio	10%	0.3 (1.1)	0.2 (0.6)	0.28	0.10	0.0043
Bacteria;__Firmicutes;__Clostridia;__Clostridiales;__Christensenellaceae;__Christensenellaceae_R-7_group	80%	1.2 (1.4)	2.1 (2.4)	-1.22	0.43	0.0048
Bacteria;__Firmicutes;__Erysipelotrichia;__Erysipelotrichales;__Erysipelotrichaceae;__Asteroleplasma	10%	0.1 (0.2)	0 (0)	0.37	0.13	0.0054
Bacteria;__Firmicutes;__Clostridia;__Clostridiales;__Ruminococcaceae;__DTU089	30%	0 (0)	0 (0)	0.82	0.29	0.0055
Bacteria;__Firmicutes;__Clostridia;__Clostridiales;__Lachnospiraceae;__Tyzzerella	10%	0 (0.1)	0 (0)	0.47	0.17	0.0060
Bacteria;__Cyanobacteria;__Melainabacteria;__Gastranaerophilales;__uncultured_bacterium;Other	10%	0.1 (0.3)	0 (0.1)	0.25	0.10	0.0087
Bacteria;__Firmicutes;__Clostridia;__Clostridiales;__Ruminococcaceae;__uncultured	100%	0.2 (0.2)	0.3 (0.2)	-0.60	0.23	0.0087
Bacteria;__Firmicutes;__Clostridia;__Clostridiales;__Ruminococcaceae;__Ruminococcaceae_UCG-013	100%	0.4 (0.2)	0.2 (0.2)	0.85	0.33	0.0090
Bacteria;__Cyanobacteria;__Melainabacteria;__Gastranaerophilales;__Clostridium_sp._K4410.MGS-306;Other	10%	0.7 (2.1)	0.3 (1)	0.27	0.10	0.0097
Bacteria;__Firmicutes;__Negativicutes;__Selenomonadales;__Veillonellaceae;__Mitsuokella	10%	0 (0.1)	0 (0.1)	0.29	0.11	0.0100
Bacteria;__Bacteroidetes;__Bacteroidia;__Bacteroidales;__Muribaculaceae;__uncultured_bacterium	10%	0.1 (0.3)	0 (0)	0.36	0.14	0.0116
Bacteria;__Firmicutes;__Clostridia;__Clostridiales;__Ruminococcaceae;__Flavonifractor	40%	0 (0)	0.1 (0.1)	-0.85	0.34	0.0130
Bacteria;__Bacteroidetes;__Bacteroidia;__Bacteroidales;__Prevotellaceae;__Prevotella_7	40%	0.1 (0.2)	0.3 (0.7)	-1.05	0.42	0.0134
Bacteria;__Proteobacteria;__Gammaproteobacteria;__Enterobacteriales;__Enterobacteriaceae;__Klebsiella	10%	0 (0)	0 (0)	0.26	0.11	0.0151
Bacteria;__Bacteroidetes;__Bacteroidia;__Bacteroidales;__Prevotellaceae;__Prevotella_2	30%	1.5 (4.9)	0.5 (1.5)	-0.96	0.40	0.0173

Bacteria;__Firmicutes;__Negativicutes;__Selenomonadales;__Acidaminococcaceae;__Acidaminococcus	10%	0 (0)	0 (0)	0.21	0.09	0.0206
Bacteria;__Firmicutes;__Clostridia;__Clostridiales;__Ruminococcaceae;__Ruminococcus_2	70%	0.9 (1.6)	1.3 (1.9)	-0.84	0.37	0.0224
Bacteria;__Firmicutes;__Clostridia;__Clostridiales;__Ruminococcaceae;__Ruminiclostridium	10%	0 (0)	0 (0)	0.75	0.33	0.0227
Bacteria;__Firmicutes;__Clostridia;__Clostridiales;__Lachnospiraceae;__[Ruminococcus]_gauvreauii_group	70%	0.1 (0.1)	0.1 (0.1)	-0.93	0.41	0.0227
Bacteria;__Firmicutes;Other;Other;Other;Other	10%	0 (0)	0 (0)	0.23	0.10	0.0248
Bacteria;__Firmicutes;__Clostridia;__Clostridiales;__Lachnospiraceae;__uncultured_bacterium	70%	0 (0)	0 (0)	0.93	0.42	0.0254
Bacteria;__Firmicutes;__Clostridia;__Clostridiales;__Lachnospiraceae;__Coprococcus_3	100%	0.2 (0.1)	0.2 (0.2)	-0.41	0.19	0.0291
Bacteria;__Firmicutes;__Clostridia;__Clostridiales;__Ruminococcaceae;Other	10%	0 (0)	0 (0)	0.17	0.08	0.0335
Bacteria;__Firmicutes;__Clostridia;__Clostridiales;__Lachnospiraceae;__Tyzzerella_3	40%	0 (0.1)	0 (0)	0.41	0.19	0.0350
Bacteria;__Bacteroidetes;__Bacteroidia;__Bacteroidales;__Marinifilaceae;__Odoribacter	70%	0.2 (0.1)	0.3 (0.2)	-1.16	0.57	0.0416
Bacteria;__Bacteroidetes;__Bacteroidia;__Bacteroidales;__Barnesiellaceae;__Coprobacter	50%	0 (0)	0 (0)	0.91	0.47	0.0497

[a] Beta coefficient (SE) and P values from linear mixed models evaluating the effect of G&C versus placebo on genera, adjusted for treatment sequence and participant body mass index.
